# Efficacy and safety of salvage radiotherapy combined with endocrine therapy in patients with biochemical recurrence after radical prostatectomy: A systematic review and meta-analysis of randomized controlled trials

**DOI:** 10.3389/fonc.2022.1093759

**Published:** 2023-01-24

**Authors:** Zhanpeng Liang, Sihong Lin, Huiqin Lai, Luzhen Li, Jiaming Wu, Huatang Zhang, Cantu Fang

**Affiliations:** Department of Oncology, Zhongshan Hospital of Traditional Chinese Medicine Affiliated to Guangzhou University of Traditional Chinese Medicine, Zhongshan, Guangdong, China

**Keywords:** salvage radiotherapy, endocrine therapy, prostate cancer, biochemical recurrence, meta-analysis

## Abstract

**Background:**

The addition of endocrine therapy to salvage radiotherapy (SRT) is expected to further improve the prognosis of patients with biochemical recurrence of prostate cancer after radical prostatectomy (RP). The quantitative synthesis of clinical outcomes of SRT combined with endocrine therapy is limited. Whether salvage radiotherapy plus endocrine therapy remains inconclusive. We performed a systematic review and meta-analysis of existing randomized controlled trials to evaluate the efficacy and safety of salvage radiotherapy combined with endocrine therapy in patients with biochemical recurrence after radical prostatectomy.

**Methods:**

A systematic search of PubMed, EMBASE, and the Cochrane library was performed for articles published between January 1, 2012 and October 10, 2022. Data were analyzed using Review Manager 5.4.1 (Cochrane Collaboration Software). Main outcome and measures included biochemical progression-free survival (bPFS), metastasis free survival (MFS), overall survival (OS), and Grade 3 or higher adverse events (3+AEs), including acute and late adverse events.

**Results:**

In this systematic review and meta-analysis, 4 randomized controlled studies enrolling 2731 male (1374 of whom received SRT combined with endocrine therapy and 1357 controls) met the inclusion criteria. SRT combined with endocrine therapy were related to significantly improve bPFS (HR=0.52; 95% CI: 0.46 0.59; p<0.00001) and MFS (HR=0.75; 95% CI: 0.64 0.88; p<0.001). Compared with SRT alone, the combination therapy tended to be associated with prolong OS (HR=0.83; 95% CI: 0.69-1.01; p=0.06), but not statistically significant. At early follow-up, the risk of acute AEs was comparable in the two groups (RR=1.04; 95% CI: 0.22-4.85). However, the risk of late AEs was higher in the combination group at later follow-up (RR=1.33; 95% CI: 1.09-1.62).

**Conclusions:**

This systematic review and meta-analysis found superior efficacy associated with adding endocrine therapy to SRT compared with SRT alone in patients with biochemical recurrence after RP. Additional endocrine therapy is safe and feasible for patients with biochemical recurrence after RP.

**Systematic review registration:**

https://www.crd.york.ac.uk/prospero, identifier (CRD42022365432).

## 1 Introduction

Prostate cancer is a leading cause of morbidity and mortality in men worldwide. Radical prostatectomy (RP) is one of the most effective treatments of localized prostate cancer. However, recurrence after RP should not be overlooked. It has been reported that 20-50% of men still experience sustained or delayed increase of prostate specific antigen (PSA) within 5-10 years after prostatectomy, which is called biochemical recurrence (1).The level of PSA has proven to be a sensitive indicator for monitoring the recurrence of prostate cancer after operation (2). The recurrence of local prostate tumor in situ, metastasis to occult lymph nodes, or distant metastasis may account for biochemical recurrence (3–5). While imaging shows no signs of recurrence (6). Thus, patients with biochemical recurrence are at greater risk of metastasis (3). Consequently, biochemical recurrence after prostatectomy is a key clinical management issue for patients with prostate cancer. As a common salvage treatment for biochemical recurrence of PSA after RP, salvage radiotherapy (SRT) should be recommended to most patients when PSA elevates after prostatectomy (7).Whereas, the efficacy of SRT is unsatisfactory. Endocrine therapy can reduce local recurrence and distant metastasis of prostate cancer after RP (8). It is reported that (9), radiotherapy combined with endocrine therapy showed good efficacy as salvage therapy for biochemical recurrence of prostate cancer. Since most of the previous studies were retrospective, the addition of endocrine therapy to salvage radiotherapy was inconclusive. At present, some results in randomized controlled studies have come out, so we performed a systematic review and meta-analysis to evaluate the efficacy and safety of SRT combined with endocrine therapy in patients with biochemical recurrence after RP.

## 2 Methods

This study was registered in the PROSPERO database (CRD42022365432) and was conducted according to the preferred reporting project for systematic review and meta-analysis (PRISMA) statement (10).And this study aims to compare the efficacy and safety of SRT combined with endocrine therapy versus SRT alone in patients with biochemical recurrence after RP.

### 2.1 Search strategy

A comprehensive search of records from January 1, 2012 to the present through the PubMed, Embase and Cochrane Library databases was carried out (date of the last search: October 10, 2022). The keywords or corresponding grid terms used to search the database are: Prostate Cancer, Radiotherapy, hormone therapy, endocrine therapy, surgery, biochemical recurrence, etc. The detailed search strategy is as described in the **eMethods** in the Supplement. Reference list of all selected articles will independently screened to identify additional studies left out in the initial search.

### 2.2 Eligibility criteria

The inclusion criteria used to select studies in this meta-analysis were (1): patients with cytologic or pathological diagnosis of prostate cancer (2) patients older than 18 years of age (3) Phase II or III prospective, randomized trials (RCTs) comparing SRT combined with endocrine therapy and SRT, (4) patients with biochemical recurrence after RP. (5) Studies reporting at least one of the following outcomes: biochemical progression-free survival (bPFS), metastasis-free survival (MFS), overall survival (OS), and grade 3 or worse adverse events (grade 3+ AEs).

The exclusion criteria were listed below: (1) SRT in combination with other therapies for prostate cancer. (2) Non-randomized controlled studies, basic studies, retrospective studies, case reports, duplicate publications, and studies where relevant data could not be extracted.

### 2.3 Study selection and data extraction

Two experienced system reviewers independently screened records for eligibility. Differences were resolved by consulting a third reviewer. Articles were retrieved from the electronic database. We screened the remaining articles for potentially eligible references by browsing titles and abstracts after removing duplicates. Then we obtained the full text of potentially eligible articles and reviewed them for eligibility.

Data was extracted using the data collection form pre-specified in Microsoft Excel. The extracted data included: baseline characteristics, sample size, interventions used, PFS, MFS, OS, and grade 3+ AEs. bPFS was defined as the time from randomization to the first occurrence of biochemical recurrence. MFS was defined as the time from randomization to the recording of metastasis or all-cause death. OS was defined as the time from randomization to death. AEs include acute AEs and late AEs. Acute AEs occurred during the treatment period and within the following 6 months. And late AEs appear after the first 6 months of treatment. Two reviewers extracted relevant data independently. If there were any differences, the two reviewers discussed and integrated the opinions of the third reviewer to decide whether to include or not. When multiple articles contained overlapping patient series, we preferentially extracted outcome data from the primary article with the largest sample size for early outcomes and the article with the longest follow-up for late outcomes.

### 2.4 Quality assessment

Tools used by the Cochrane Collaboration to assess the risk of bias in trials were used, including the following areas: random sequence generation, allocation concealment, blinding, incomplete outcome data, and selective outcome reporting (11). Two reviewers independently assessed trial quality and resolved differences by consulting a third reviewer.

### 2.5 Statistical analysis

Data were analyzed using Review Manager 5.4.1 (Cochrane Collaboration Software). bPFS, MFS, and OS were reported as hazard ratios (HR) with corresponding 95% confidence intervals (95%CI), and Grade 3+ AEs were reported as hazard ratios with 95%CI. P value <0.05 was considered statistically significant. For effectiveness and side effects, HR or RR < 1 favored SRT alone group (control), while HR or RR < 1 favored SRT combined with endocrine therapy (experimental). Heterogeneity was tested using the I² statistic. If I²≤50%, the fixed effects model is used, and if I²>50%, using a random effects model pool the data (12, 13). Egger regression test and funnel plot were used to evaluate publication bias (14).

## 3 Results

### 3.1 Study identification and quality assessment

**Figure 1:**
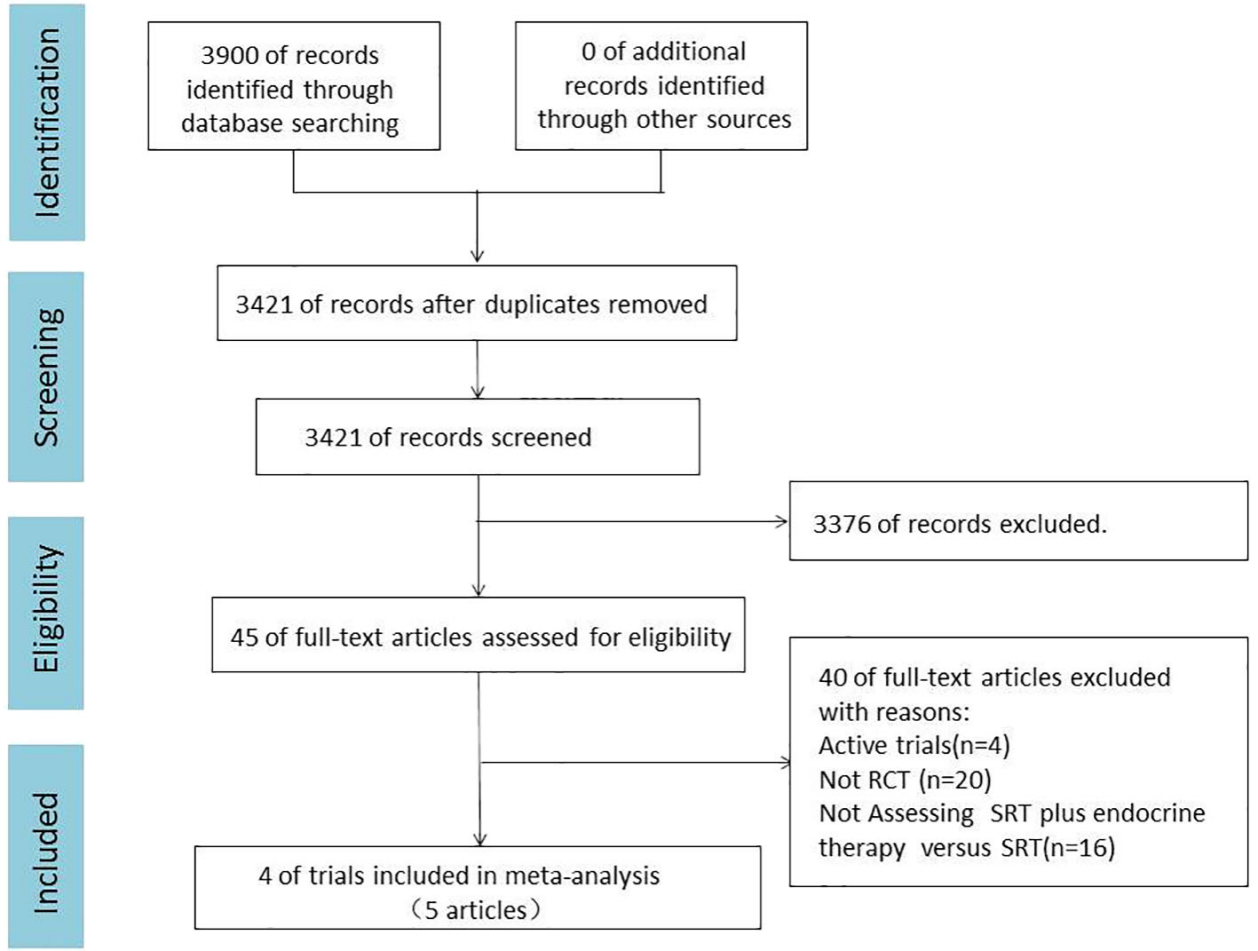
PRISMA Flow Diagram. RCT, randomized controlled trial; SRT, salvage radiotherapy.

A total of 3900 articles were retrieved from three electronic databases: PubMed, EMBASE and Cochrane library. After removing 479 duplicates, 3376 papers were excluded by reading the title and abstract. 45 full-text articles were reviewed. Finally, this meta-analysis included 4 randomized controlled trials (5 publications) involving 2731 patients (15–19). A PRISMA flow diagram depicting the study identification and selection is shown in **Figure 1**. The primary sources of bias owed to differences in the definition of biochemical recurrence and a lack of blinding in some trials (**eTable 3** in the Supplement).

### 3.2 Study and patient characteristics

All four studies reported detailed data on bPFS. Three trials provided detailed data on MFF and OS. Three studies reported adverse events (**Table 1**). The characteristics of the 4 trials are reported in **eTables 1**, **2** in Supplement. SPPORT (17) enrolled 1792 eligible patients in the United States, Canada, and Israel from March 31, 2008 to March 30, 2015, and RTOG 9601 (18) enrolled 840 patients in the United States and Canada from March 1998 to March 2003. GETUG-AFU 16 (16) recruited 743 patients in France from October 19, 2006 to March 30, 2010. SALV-ENZA (15) began its study on March 28, 2015, with the aim of enrolling 96 patients in the United States. Survival data from 86 patients in this trial were presented at the 2022 ASCO annual meeting.

**Table 1:** Summary of efficacy end points and adverse events.

Trail	bPFS	MFS	OS	Grade 3+ acute AEs (%)		Grade 3+ late AEs (%)	
	HR (95% CI)	HR (95% CI)	HR (95% CI)	Endocrine Therapy	Control	Endocrine Therapy	Control
RTOG 9601^(18,19)^	0.48 (0.40-0.58)	0.73 (0.58-0.92)	0.77 (0.59-0.99)	2.1	4.85	26.2	19.5
GETUG-AFU 16^(16)^	0.54 (0.43-0.68)	0.73 (0.54-0.98)	0.93 (0.63-1.39)	NA	NA	NA	NA
SPPORT^(17)^	0.59 (0.47-0.74)	0.82 (0.60-1.13)	0.89 (0.60-1.31)	7.3	3.3	15.5	11.6
SALV-ENZA^(15)^	0.40 (0.17-0.92)	NA	NA	NA	NA	NA	NA

bPFS, biochemical progression-free survival; MFS, metastasis-free survival; OS, overall survival; grade 3+,grade 3 or worse; AEs, adverse events; HR, hazard ratio; CI, confidence interval; NA, not available.

All four trials evaluated the effect of adding endocrine therapy to SRT on the prognosis of patients with biochemical recurrence of prostate cancer after surgery. SPPORT (17) additionally assessed the efficacy of SRT combined with endocrine therapy and pelvic lymph node radiotherapy (PLNRT). Endocrine therapy regimens differed among the four studies (**eTable 4** in the Supplement). SPPORT (17) uses a 4-6 month regimen of luteinizing hormone-releasing hormones (LHRH) and antiandrogens (AA). Patients in GETUG-AFU 16 received goserelin for 6 months (16). Patients in SALV-ENZA were maintained with enzalutamide for 6 months (15). Bicalutamide was administered with RTOG 9601 for 2 years (18). Four trials included prostate cancer patients with localized lesions, with similar, but non-identical, definitions. GETUG-AFU 16 (16), RTOG 9601 (18) and SPPORT (17) all included males in T2 and T3. However, GETUG-AFU 16 16additional patients received T4a (bladder neck involvement only). For SALV-ENZA, the aim was to enroll high-risk patients including those with Gleason score 8–10 or 7 and either pT3 or positive margins.

RTOG 9601 (18) and GETUG-AFU 16 (16) triggered SRT at a PSA level of 0.2ng/ml, while a PSA level of 0.1ng/ml was required for SPPORT (17) and SALV-ENZA (15). The definition of biochemical recurrence after randomization also differed slightly between the included trials (**eTable 4** in the Supplement).In RTOG 9601 (18), biochemical recurrence was defined as an increase in PSA concentration of more than 0.3-0.5ng/mL from the lowest value. In GETUG-AFU 16 (16), an increase of more than 0.5ng/mL from the lowest value was required to meet the criteria. And for SPPORT (17) and SALV-ENZA (15), biochemical recurrence was defined as an increase in PSA concentration of more than 0.4ng/mL from the lowest value.

### 3.3 Results of meta-analysis

#### 3.3.1 Biochemical progression-free survival

**Figure 2:**
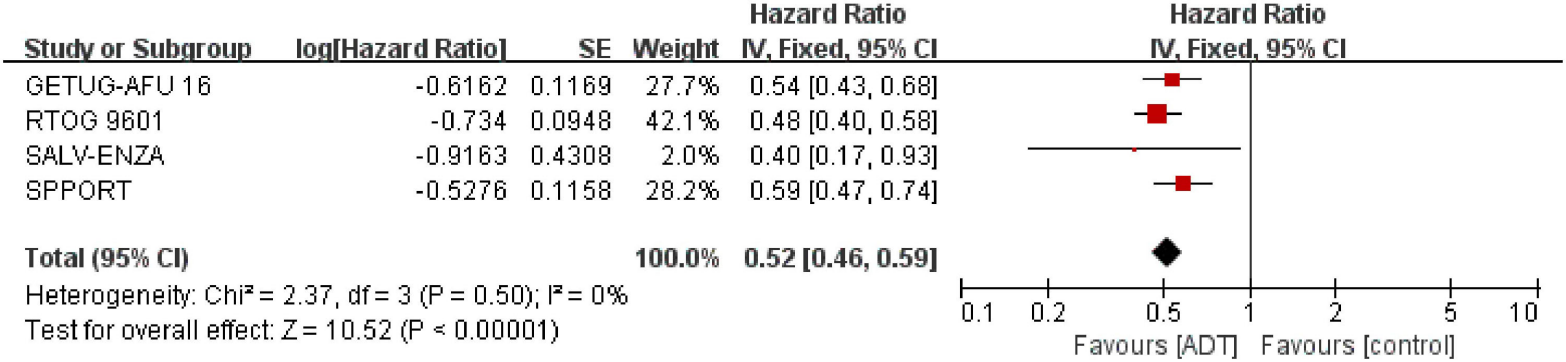
Assessment of biochemical Progression Free Survival. The diamond indicates best estimate of the true (pooled) outcome (with width indicating 95% CI); HR, hazard ratio; experimental stands for salvage radiotherapy combined with endocrine therapy; control stands for salvage radiotherapy alone. Since there is no heterogeneity, a fixed-effects model is used.

We extracted the results of bPFS from 2731 patients from four trials (15–18). Overall, patients receiving SRT plus endocrine therapy resulted in longer biochemical progression-free survival (HR=0.52; 95% CI: 0.46-0.59; p < 0.00001), with no heterogeneity (Chi^2^ = 2.37; df = 3 [p= 0.50]; I^2^ = 0%, **Figure 2**). We performed subgroup analyses of PSA levels that trigger SRT. Whether SRT was triggered at a PSA level of 0.1 or 0.2ng/ml, the results favored combination therapy (**eFigure 1** in the Supplement).

#### 3.3.2 Metastasis-free survival

**Figure 3:**
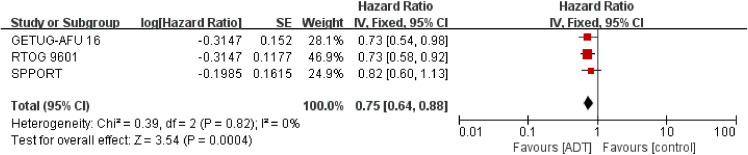
Assessment of Metastasis Free Survival. The diamond indicates best estimate of the true (pooled) outcome (with width indicating 95% CI); HR, hazard ratio; experimental stands for salvage radiotherapy combined with endocrine therapy; control stands for salvage radiotherapy alone. Since there is no heterogeneity, a fixed-effects model is used.

Detailed data of MFS were extracted from 2645 patients in three trials (16, 17, 19). The results showed that SRT combined with endocrine therapy had a significant benefit in MFS compared with SRT alone. (HR = 0.75; 95% CI: 0.64 0.88; p=0.0004), with no heterogeneity (Chi^2^ = 0.39; df = 2 [p= 0.82]; I^2^ = 0%, **Figure 3**). Analysis of trials triggering SRT at a PSA level of 0.2ng/ml showed similar results to the total effect value (**eFigure 2** in the Supplement).

#### 3.3.3 Overall survival

**Figure 4:**
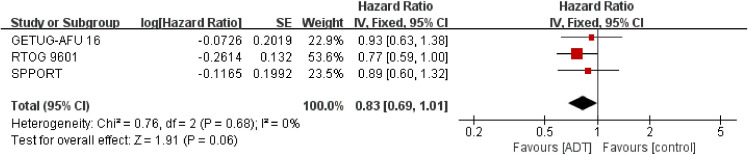
Assessment of Overall Survival. The diamond indicates best estimate of the true (pooled) outcome (with width indicating 95% CI). HR, hazard ratio; experimental stands for salvage radiotherapy combined with endocrine therapy; control stands for salvage radiotherapy alone. Since there is no heterogeneity, a fixed-effects model is used.

Analysis of OS, which included 2645 patients in three trials (16–18), showed that SRT combined with endocrine therapy tended to be associated with prolong OS (HR = 0.83; 95% CI: 0.69-1.01; P = 0.06), but the difference was not statistically significant, with no heterogeneity (Chi_2_ = 0.76; df = 2 [p= 0.68]; I_2_ = 0%, **Figure 4**). Analysis of trials triggering SRT at a PSA level of 0.2ng/ml showed similar results to the total effect value (**eFigure 3** in the Supplement).

#### 3.3.4 Adverse events

Adverse events, including acute and late AEs, were extracted from three studies (16–18). Endocrine therapy in all three studies was initiated immediately after the initiation of radiation therapy, so endocrine therapy may have a potential impact on both acute and chronic AEs. SRT combined with endocrine therapy did not result in a higher incidence of grade 3+ acute AEs (RR=1.04; 95% CI: 0.22-4.85; p=0.96), but resulted in more severe grade 3+ late AEs (RR=1.33; 95% CI: 1.09-1.62; P = 0.006, **eFigures 4**, **5** in the Supplement). There was no significant difference between SRT combined with endocrine and SRT alone in renal or genitourinary, hematologic and gastrointestinal toxicities, regardless of acute or late AEs. But the combination of SRT and endocrine therapy led to more severe sexual dysfunction (**Table 2**).

**Table 2:** Results of Acute And Late Adverse Avents.

	Acute AEs	No. of trials	Late AEs	No. of trials
	RR (95% CI)		RR (95% CI)	
Renal or genitourinary	0.57(0.21-1.53)	2	1.06(0.80-1.41)	3
Hematologic	0.81(0.19-3.74)	2	1.12(0.47-3.07)	2
Any GI AEs	1.29(0.09-18.27)	2	1.36(0.72-2.58)	3
Sexual disorder	NA	NA	1.61(1.08-2.41)	2
Any AE grade 3+	1.04(0.22-4.85)	2	1.32(1.09-1.62)	2

GI, gastrointestinal; grade 3+, grade 3 or worse; AE, adverse event; RR, risk ratio; CI, confidence interval; NA, not available.

### 3.4 Heterogeneity and publication bias

The only outcome for which we identified significant heterogeneity among studies was acute toxicity. The low incidence of acute toxicity may account for the heterogeneity. All other results showed no heterogeneity, with the I^2^ value of 0%. The overall quality of the included studies was high. Two of the included RCTs were open-label, with some risk of bias. Because all trials have been properly randomised, the risk of confounding is minimal in RCTs. Funnel plot asymmetry is not obvious to any result (**eFigures 6**–**8** in the Supplement). Egger regression test results showed that bPFS (P=0.786), MFS (P=0.676) and OS (P=0.097) had a low possibility of publication bias.

## 4 Discussion

Serum PSA is considered as one of the most sensitive tumor markers. Therefore, for early detection of prostate cancer, PSA screening is reckoned as an effective method to reduce prostate cancer mortality (20), for biochemical recurrence seems to be the only evidence of disease progression in the absence of clinical symptoms. It is worth noting that bout a third of patients experience biochemical recurrence after RP, depending on their initial prognosis (1) which entails a comprehensive assessment of local and systemic recurrence so as to carry out active intervention. SRT has proven to enhance the outcomes of patients with biologically recurrent prostate cancer (7). But whether to add endocrine therapy to radiotherapy still remains controversial. This meta-analysis summarized randomized controlled studies evaluating the effect of SRT combined with endocrine therapy on postoperative biochemical recurrence of prostate cancer. The results indicated that SRT combined with endocrine therapy significantly improved bPFS and MFS, with a trend of benefit in the term of OS, even though the difference was not statistically significant.

A previous meta-analysis (21) involving 12 studies also showed a significant benefit of SRT combined with endocrine therapy for bPFS. However, most of the studies included in this meta-analysis were retrospective, with only two RCTs, so the credibility of the conclusion was very low. Our meta-analysis, which included only randomized controlled studies, not only updated the latest results of two previous RCTs, but also included two new studies. Preclinical studies have found superadditive effects of the combination of radiotherapy and endocrine therapy (22), suggesting a potential benefit of the combination. This was confirmed by clinical results, with all four included studies indicative of benefit for bPFS, which was in accordance with our findings.

As the first study to evaluate the effect of adding endocrine therapy to SRT on the prognosis of patients with biochemical recurrence after RP, the RTOG 9601 trial (18) revealed that SRT plus 24 months of bicalutamide anti-androgen therapy significantly improved long-term OS after preforming an analysis of 760 patients. SPPORT (17) and GETUG-AFU 16 (16) did not show significant benefits for OS. It is noteworthy that, endocrine therapy in SPPORT (17) and GETUG-AFU 16 (16) lasted no more than 6 months, whereas anti-androgen therapy in RTOG 9601 (18) lasted 24 months, which may account for the difference. The RADICALS-HD trial, whose results have not been released, enrolled 3000 men undergoing postoperative prostate bed radiotherapy and compared SRT alone versus SRT plus 6 months of ADT versus SRT plus 24 months of ADT (NCT00541047). The results of that trial are expected to provide more valuable guidance for clinical practice. In addition, longer follow-up is needed to determine the impact of interventions on OS in patients with biochemical recurrence after RP. The incidence of death events was low in all three studies, so follow-up time was also a factor affecting OS. As can be seen, SPPORT (17) and GETUG-AFU 16 (16) also showed a trend of prolonged OS, though it was not statistically significant. Our review indicates a similar result. In this case, MFS is seen as a good surrogate end point for OS. Xie et al. (23) collected individual patient data from 28905 patients from 28 trials. At the patient level, Kendall’s tau coefficient correlation with OS was 0.91 for MFS, demonstrating that MFS was a strong surrogate for OS in localized prostate cancer and was significantly associated with the risk of death from prostate cancer. Given the strong correlation between MFS and OS, it makes perfect sense to use MFS as the primary end point instead of OS. In our review, SRT plus endocrine therapy resulted in longer MFS, suggesting that this combination therapy is related to better prognosis of patients with biochemical recurrence after RP.

The four included trials did not fully agree on the definition of biochemical recurrence. RTOG 9601 (18) and GETUG-AFU 16 (16) triggered SRT at a PSA level of 0.2ng/ml, while a PSA level of 0.1ng/ml was required for SPPORT (17) and SALV-ENZA (15). Therefore, subgroup analyses were performed to explore this difference. The results showed that the definition of biochemical recurrence before treatment had no effect on bPFS. In terms of MFS and OS, the results of the subgroup triggering SRT at 0.2ng/ml PSA level were consistent with the overall effect. The definition of biochemical recurrence after treatment was also slightly different. However, a Japanese study reported that this difference contributed to the overall effect difference, but the effect was small (24). This study reviewed the data of 118 patients who underwent SRT for biochemical recurrence after RP. The 2-year bPFS rates defined using Nara, RTOG 9601 and GETUG-AFU 16 were 59%, 70% and 69%, respectively. In addition, all four trials had a clear benefit with respect for bPFS which seemed unaffected by differences in definition.

The baseline characteristics of prostate cancer patients have potential impact to the effect of endocrine therapy. In a recently published meta-analysis, multiple analyses involving androgen-receptor-axis-targeted (ARAT) based treatment were conducted by pooling the PFS- and OS-HRs of different between subgroups defined according to different baseline characteristics, with the intent to identify predictors of efficacy in ARAT therapies for metastatic castration-sensitive prostate cancer (25). The authors concluded that prior docetaxel use and tumor volume/presence of visceral metastasis were the only factors that negatively affected the efficacy of ARAT, which seems to indicate that metastatic prostate cancer with a low tumor burden may benefit more from ARAT treatment. However, patients with pT3 localized prostate cancer in the SALV-ENZA trial benefited more significantly from enzulamide than patients with pT2 (interaction p =0.031) (15). Similarly, the subgroup analysis of the RTOG 9601 trial found the greatest overall survival benefit in the subgroup of patients with more aggressive prostate cancer (18). In another meta-analysis, an overall survival benefit from hormone therapy was found in RTOG 9601 trial limited to follow-up extended to ≥10 years, pre-SRT PSA ≥0.7ng/ml, or the presence of a higher gleason grade or positive margin (26). In the secondary analysis of RTOG 9601, higher pre-SRT PSA levels (PSA>0.6ng/mL) and higher Decipher scores were associated with greater benefit of hormone therapy (27, 28). Previous studies have found that providing SRT in lower PSA levels is associated with improved outcomes (29, 30). As a result, men with low PSA levels get less absolute benefit from hormone therapy. Patients with higher PSA levels are more likely to benefit from ADT, possibly because additional ADT can eliminate radiation-offsite subclinical metastases. In contrast, the insufficient effect of positive margins on the risk of metastasis may be related to higher dose administration and more timely use of SRT, which may lead to better local disease control (26, 31, 32). A cohort study found that ADT was beneficial only in patients with more aggressive traits, namely, pT3b/4 and grade group>4 or pT3b/4 and PSA at eSRT>0.4 ng/ml (8), which is recommended by the current csco guidelines. In our analysis of these limited clinical trials, we found that patients with more aggressive prostate cancer (including high baseline PSA, gleason score ≥ 8, and pT3b/4) were more likely to benefit from endocrine therapy. Unfortunately, we failed to further explore the effect of baseline characteristics on endocrine therapy in patients with prostate cancer due to insufficient data for subgroup analyses. So these conclusions are still premature and need to be confirmed by more clinical trials.

Exploring prognostic factors helps establish accurate risk stratification. GETUG-AFU 16 found that baseline PSA levels, surgical margin status, PSA doubling time at recurrence, and seminal vesicle status had a negative impact on disease progression (16, 33). Moreover SPPORT found that baseline PSA levels, seminal vesicle involvement, and Gleason score were prognostic factors for disease progression (17). Heterogeneity of prostate cancer is one of the common reasons of biochemical recurrence after RP, so better stratification of prostate cancer patients from diagnosis to early biochemical recurrence is essential. However, current risk stratification models are limited. An emerging field of research is helping us solve this problem how to more accurately describe the prognosis for each patient and each stage of the disease. As a rapidly growing field of research, radiomics features (RFs) analysis transforms visual image information into in-depth features for quantitative study that can inform detection, risk stratification, and treatment (34). For example, due to its very high sensitivity and specificity, prostate-specific membrane antigen positron emission tomography (PSMA-PET) can improve the detection of biochemical recurrence and metastasis, especially at low PSA levels (35), which has been difficult to achieve with previous detection methods. PSMA-PET can identify patients who need additional lymph node radiotherapy or ADT by locating the lymph node recurrence in patients with biochemical recurrence with aims to provide more personalized treatment (36–38). Studies have found that SRT guided by PSMA-PET has a better prognosis than non-PSMA-PET guided SRT (39). In addition, the artificial intelligence (AI) and machine-learning (ML) techniques are attractive research fields in prostate cancer. With high predictive accuracy, AI and ML can integrate medical imaging, genetic and clinical big data to obtain more information about the molecular and biological aspects of prostate cancer (34). By combining RFs with AI and ML methods, these applications could hold promise for personalized and precision therapy in the future.

For adverse effects, this meta-analysis found that endocrine therapy did not induce more severe grade 3+ acute toxicities, but would increase the risk of overall grade 3+ chronic toxicities. Among the common chronic toxicities, compared with placebo, endocrine therapy induced higher severity of sexual dysfunction, without increasing the incidence of renal or genitourinary, hematologic and gastrointestinal toxicities. Endocrine treatment-related cardiotoxicity is a common concern because severe cardiotoxicity can be fatal. In the RTOG 9601 trial, cardiac events were 17 in the bicalutamide group and 6 in the placebo group (18). The secondary analysis of RTOG 9601 found that an increase in other-cause mortality (OCM) from the primary cause of early PSA was seen in low PSA (0.2 to 0.3 ng/mL) patients, who had a 13.3% reduction in 12-year OS (27). LHRH agonists and GNRH antagonists also lead to higher rates of cardiac events, but the rates of cardiovascular events are somewhat different (40). A meta-analysis found that degarelix was associated with a lower incidence of cardiovascular events compared with LHRH agonists (41). But degarelix had a higher incidence of cardiovascular events in patients with preexisting cardiovascular disease (42). Overall, long-term antiandrogen therapy is associated with increased cardiovascular events and mortality, which may offset the increased potential survival in patients with low pre-SRT PSA.

This meta-analysis has several strengths including the prospective registration of the systematic review protocol and no heterogeneity in any efficacy end point. Additionally, to eliminate the limitation of follow-up time, we pooled MFS data with the aim of attempting to replace OS to evaluate the efficacy of endocrine therapy and showed that additional endocrine therapy could improve MFS. Moreover, our review represents the full range of randomized evidence for the effect of adding endocrine therapy to SRT in patients with biochemical recurrence after RP. There are also limitations in our study. Firstly, only 4 trials were included in our review. Secondly, there were some differences in the definitions of biochemical recurrence before and after treatment among the included trials. Thirdly, it is difficult to explore the effect of baseline characteristics on endocrine therapy for lacking enough data to perform subgroup analyses of baseline characteristics in prostate cancer patients. Fourthly, the follow-up time of most studies was insufficient, making it difficult to evaluate the effect of endocrine therapy on OS more comprehensively, leading to certain deviations.

## 5 Conclusions

MFS is a strong surrogate of overall survival in localized prostate cancer. This meta-analysis found superior bPFS and MFS associated with additional endocrine therapy in patients with biochemical recurrence after RP. Although the follow-up time was limited, there was also a trend of benefit in the term of OS. Therefore, adding endocrine to SRT is a reasonable and necessary. But more high-quality studies are needed to determine the duration and specific drugs of endocrine therapy. Patient, tumor, and treatment factors should be considered when using hormone therapy, as individualized therapy is the current trend.

## Data availability statement

The datasets presented in this study can be found in online repositories. The names of the repository/repositories and accession number(s) can be found in the article/**Supplementary Material**.

## Author contributions

ZL, SL, HL, and CF conceived and guide the study. ZL and SL performed statistical analysis, and drafted the manuscript. HZ, LL, and JW collected the literature, edited figures and revised the manuscript. All authors contributed to the article and approved the submitted version.
