# Supplementary material for: Efficacy and safety of salvage radiotherapy combined with endocrine therapy in patients with biochemical recurrence after radical prostatectomy: A systematic review and meta-analysis of randomized controlled trials
Source: Front Oncol. 2023 Jan 24;12:1093759. doi: 10.3389/fonc.2022.1093759 (PMC9902708; doi:10.3389/fonc.2022.1093759)
Supplement: Supplementary file 1 [file DataSheet_1.pdf]

Supplementary

Context:

eTable 1. Characteristics of Included Studies
eTable 2. Patient Characteristics of Included Studies
eTable 3. Cochrane Risk of Bias Assessment Among Individual Studies
eTable 4. Definition of Biochemical Recurrence Before And After Treatment
eFigure 1. Assessment of Subgroup Analysis for biochemical Progression Free Survival Based on Baseline PSA Levels.
eFigure 2. Assessment for Subgroup (triggering SRT at 0.2ng/ml PSA level) of Metastasis Free Survival
eFigure 3. Assessment for Subgroup (triggering SRT at 0.2ng/ml PSA level) of Overall Survival
eFigure 4. Assessment of Acute Adverse Events
eFigure 5. Assessment of Late Adverse Events
eFigure 6. Funnel plot of biochemical Progression Free Survival.
eFigure 7. Funnel plot of Metastasis Free Survival.
eFigure 8. Funnel plot of Overall Survival.
eMethods. Search Strategy
eReferences

This supplementary material has been provided by the authors to give readers additional information about their work.

eTable 1. Characteristics of Included Studies

Trail	Accrual period	Key eligibility criteria	endocrine therapy	salvage Radiotherapy(SRT) schedule	Primary outcome measure
**RTOG 9601**[1,2]	1998-03 to 2003-03	pT2 (positive surgical margin) or T3 (extension beyond the prostatic capsule)	Bicalutamide for 24 months	64.8 Gy (prostatic bed)	Overall survival
**GETUG-AFU 16**[3]	2006-10 to 2010-03	pT2, T3, and T4a (bladder neck involvement only)	Goserelin for 6 months	66 Gy (prostatic bed) / 46 Gy (Pelvis with lymph nod involvement)	Progression-free survival
**SPPORT**[4]	2008-03 to` 2015-03	pT2 or pT3, Gleason score of 9 or less	AA and LHRH agonist for 4-6 months	64.8-70.2 Gy (prostatic bed)	Freedom from progression
**SALV-ENZA**[5]	2015-04 to 2020-02-	Gleason score 8–10 or 7 and either pT3 or positive margins	Enzalutamide for 6 months	66.6-70.2 Gy (prostatic bed)	Freedom of PSA progression

Abbreviations: PSA: Prostate Specific Antigen; AA, antiandrogens; LHRH, luteinizing hormone-releasing hormones; NA, not available; Gy, Gray.

eTable 2. Patient Characteristics of Included Studies

	RTOG 9601[1,2]		GETUG-AFU 16[3]		SPPORT[4]		SALV-ENZA[5]	
	Endocrine Therapy	Control	Endocrine Therapy	Control	Endocrine Therapy	Control	Endocrine Therapy	Control
**Patients randomised**	384	376	369	373	578	564	43	43
**Median follow up (interquartile range)**	13years		8.2years(6.6-9.4)		9.3years(8.5-10.3)		2.8years	
**median Age (range)**	65(40-83)	65(45-81)	67(49–80)	67(52–85)	64(39–80)	64(42–84)	69(51-82)	66(52-81)
**Median pre-operative PSA (interquartile range)**	0.6 (0.2-1.0)	0.6 (0.2-1.2)	0·3 (0·2-0·5)	0·3 (0·2–0·5)	0·40(0·23-0·68)	0·32(0·20-0·60)	0.3	0.3
**Race or ethnic group, n, (%)**			NA	NA				
**White**	344 (90%)	324 (86%)			482(83%)	464(82%)	37(86%)	40 (93%)
**Black**	28 (7%)	40 (11%)			69(12%)	73(13%)	5 (12%)	3 (7%)
**Asian**	5 (1%)	4 (1%)			6(1%)	3(1%)	0	0
**Other**	7(2%)	8(2%)			21(4%)	24(4%)	1 (2%)	0
**Stage**								
**P2**	128(33%)	120(32%)	196(53%)	201(54%)	317(55%)	292(52%)	14 (33%)	15 (35%)
**P3**	256(67%)	256(68%)	171(46%)	171(45%)	261(45%)	272(48%)	28 (65%)	28 (65%)
**P4**	0	0	1(<1%)	0	0	0	0	0
**Unkonw**	0	0	1(<1%)	1(<1%)	0	0	1(2%)	0
**Gleason score**								
**≤7**	316(83%)	311(83%)	329(89%)	322(89%)	480(83%)	471(84%)	25(58%)	22(51%)
**≥8**	67(17%)	64(17%)	40(11%)	41(11%)	98(17%)	93(16%)	18(42%)	21(49%)
**Unkonw**	1(<1%)	1(<1%)	0	0	0	0	0	0
**Positive margin**								
**Yes**	96(25%)	95(25%)	175(47%)	196(53%)	289(50%)	288(51%)	22 (51%)	21(19%)
**No**	288(75%)	281(75%)	194(53%)	177(47%)	284(49%)	267(47%)	21(19%)	22 (51%)
**Unkonw**			0	0	5(1%)	9(2%)	0	0
**Seminal vesicle involvement**	NA	NA					NA	NA
**Yes**			312(85%)	318(85%)	494(86%)	482(86%)		
**No**			57(15%)	55(15%)	84(14%)	82(14%)		
**Unknow**			0	0				

The SALV-ENZA did not report the specifics of the experimental and control groups, so only the overall population is described.
Abbreviations: PSA, Prostate Specific Antigen; NA, not available.

eTable 3. Cochrane Risk of Bias Assessment Among Individual Studies

Domain	RTOG 9601[1,2]	GETUG-AFU 16[3]	SPPORT[4]	SALV-ENZA[5]
**Random Sequence Generation**	Low risk	Low risk	Low risk	Low risk
**Allocation Concealment**	unclear	unclear	unclear	unclear
**Blinding of Participants**	Low risk	High risk	High risk	Low risk
**Blinding of Personnel**	Low risk	High risk	High risk	Low risk
**Blinding of Outcome Assessment**	Low risk	unclear	unclear	unclear
**Incomplete Outcome Data**	Low risk	Low risk	Low risk	unclear
**Selective Outcome Reporting**	Low risk	Low risk	Low risk	Low risk
**Other Sources of Bias**	High risk	High risk	High risk	High risk
**Other Sources of Bias Description**	the definitions of biochemical recurrence before and after treatment difference from other trails	the definitions of biochemical recurrence before and after treatment difference from other trails	the definitions of biochemical recurrence before and after treatment difference from other trails	the definitions of biochemical recurrence before and after treatment difference from other trails

eTable 4. Definition of Biochemical Recurrence Before And After Treatment

Trail	Baseline PSA level before treatment (range)	Definition of biochemical recurrence after treatment
**RTOG 9601[1,2]**	0·2–4ng/ml (at least 12 weeks after prostatectomy)	If PSA <0.2ng/ml during PT, failure = increase after PT to≥0.5ng/ml ;<br>If PSA≥ 0.2ng/ml during PT, failure = increase PT in PSA after of≥0.3ng/ml above the lowest detectable level;<br>If PSA did not decrease during PT, failure = increase in PSA after PT of≥0.5ng/ml above entry PSA level.
**GETUG-AFU 16[3]**	0·2–2ng/mL (at least 6 months after prostatectomy)	PSA concentration above the nadir of ＞0·5ng/L
**SPPORT[4]**	0.1-2.0ng/mL (at least 6 weeks after prostatectomy)	PSA concentration above the nadir of ≥0·4ng/L
**SALV-ENZA[5]**	0.05-0.7ng/mL	PSA concentration above the nadir of ≥0·2ng/L that was confirmed by a second consecutive PSA value obtained ≥ 8 weeks later which was higher (and ≥ 0.4 ng/mL).

Abbreviations: PSA, Prostate Specific Antigen; PT, protocol treatment;

eFigure 1. Assessment of Subgroup Analysis for biochemical Progression Free Survival Based on Baseline PSA Levels.

Study or Subgroup	log[Hazard Ratio]	SE	Weight	Hazard Ratio IV, Fixed, 95% CI
**1.6.1 PSA ≥0.1ng/ml**				
SALV-ENZA	-0.9163	0.4308	6.7%	0.40 [0.17, 0.93]
SPPORT	-0.5276	0.1158	93.3%	0.59 [0.47, 0.74]
**Subtotal (95% CI)**			**100.0%**	**0.57 [0.46, 0.72]**
Heterogeneity: Chi² = 0.76, df = 1 (P = 0.38); I² = 0%				
Test for overall effect: Z = 4.95 (P < 0.00001)				
**1.6.2 PSA ≥0.2ng/ml**				
GETUG-AFU 16	-0.6162	0.1169	39.7%	0.54 [0.43, 0.68]
RTOG 9601	-0.734	0.0948	60.3%	0.48 [0.40, 0.58]
**Subtotal (95% CI)**			**100.0%**	**0.50 [0.44, 0.58]**
Heterogeneity: Chi² = 0.61, df = 1 (P = 0.43); I² = 0%				
Test for overall effect: Z = 9.33 (P < 0.00001)				
Test for subgroup differences: Chi² = 0.99. df = 1 (P = 0.32). I² = 0%				

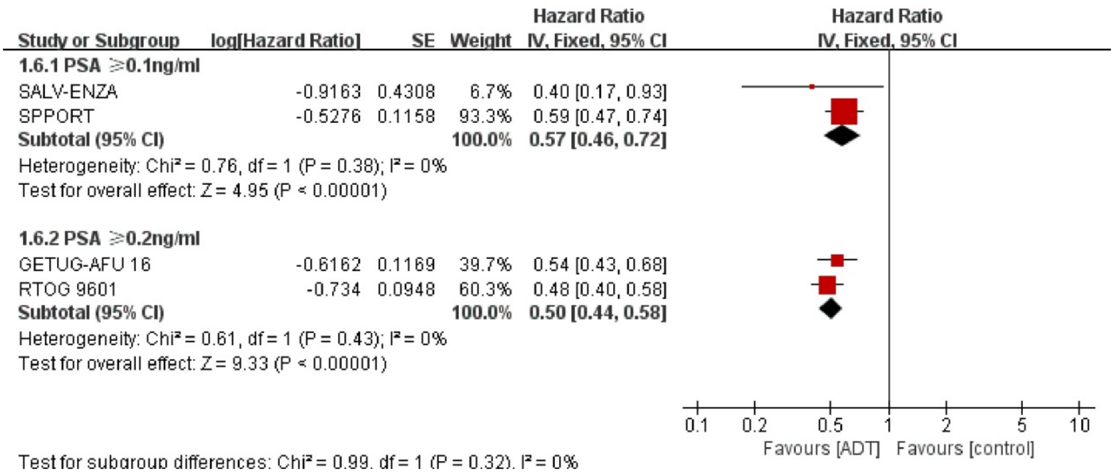

Abbreviations: The diamond indicates best estimate of the true (pooled) outcome (with width indicating 95% CI). HR, hazard ratio; experimental stands for salvage radiotherapy combined with endocrine therapy; control stands for salvage radiotherapy alone. Since there is no heterogeneity, a fixed-effects model is used.

eFigure 2. Assessment for Subgroup (triggering SRT at 0.2ng/ml PSA level) of Metastasis Free Survival

Study or Subgroup	log[Hazard Ratio]	SE	Weight	Hazard Ratio IV, Fixed, 95% CI
GETUG-AFU 16	-0.3147	0.152	37.5%	0.73 [0.54, 0.98]
RTOG 9601	-0.3147	0.1177	62.5%	0.73 [0.58, 0.92]
**Total (95% CI)**			**100.0%**	**0.73 [0.61, 0.88]**
Heterogeneity: Chi² = 0.00, df = 1 (P = 1.00); I² = 0%				
Test for overall effect: Z = 3.38 (P = 0.0007)				

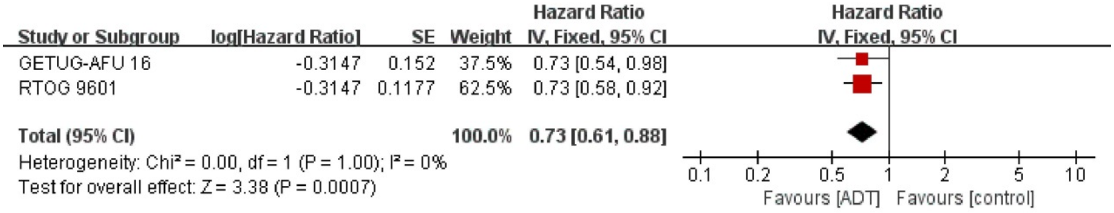

Abbreviations: The diamond indicates best estimate of the true (pooled) outcome (with width indicating 95% CI). HR, hazard ratio; experimental stands for salvage radiotherapy combined with endocrine therapy; control stands for salvage radiotherapy alone. Since there is no heterogeneity, a fixed-effects model is used.

eFigure 3. Assessment for Subgroup (triggering SRT at 0.2ng/ml PSA level) of Overall Survival

Study or Subgroup	log[Hazard Ratio]	SE	Weight	Hazard Ratio IV, Fixed, 95% CI
GETUG-AFU 16	-0.0726	0.2019	29.9%	0.93 [0.63, 1.38]
RTOG 9601	-0.2614	0.132	70.1%	0.77 [0.59, 1.00]
**Total (95% CI)**			**100.0%**	**0.81 [0.66, 1.01]**
Heterogeneity: Chi² = 0.61, df = 1 (P = 0.43); I² = 0%				
Test for overall effect: Z = 1.85 (P = 0.06)				

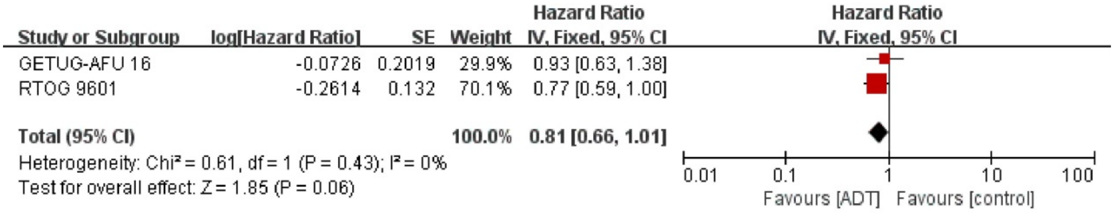

Abbreviations: The diamond indicates best estimate of the true (pooled) outcome (with width indicating 95% CI). HR, hazard ratio; experimental stands for salvage radiotherapy combined with endocrine therapy; control stands for salvage radiotherapy alone. Since there is no heterogeneity, a fixed-effects model is used.

eFigure 4. Assessment of Acute Adverse Events

Study or Subgroup	Experimental Events	Experimental Total	Control Events	Control Total	Weight	Risk Ratio M-H, Random, 95% CI
**1.4.1 Renal or genitourinary**						
RTOG 9601	4	382	11	374	53.0%	0.36 [0.11, 1.11]
SPPORT	5	563	5	547	47.0%	0.97 [0.28, 3.34]
**Subtotal (95% CI)**		**945**		**921**	**100.0%**	**0.57 [0.21, 1.53]**
Total events	9		16			
Heterogeneity: Tau² = 0.14; Chi² = 1.38, df = 1 (P = 0.24); I² = 28%						
Test for overall effect: Z = 1.12 (P = 0.26)						
**1.4.2 Hematologic**						
RTOG 9601	3	382	2	374	60.9%	1.47 [0.25, 8.74]
SPPORT	1	563	3	547	39.1%	0.32 [0.03, 3.10]
**Subtotal (95% CI)**		**945**		**921**	**100.0%**	**0.81 [0.19, 3.47]**
Total events	4		5			
Heterogeneity: Tau² = 0.07; Chi² = 1.06, df = 1 (P = 0.30); I² = 6%						
Test for overall effect: Z = 0.28 (P = 0.78)						
**1.4.3 Any GI AEs**						
RTOG 9601	1	382	3	374	49.1%	0.33 [0.03, 3.12]
SPPORT	5	563	1	547	50.9%	4.86 [0.57, 41.45]
**Subtotal (95% CI)**		**945**		**921**	**100.0%**	**1.29 [0.09, 18.27]**
Total events	6		4			
Heterogeneity: Tau² = 2.40; Chi² = 2.90, df = 1 (P = 0.09); I² = 66%						
Test for overall effect: Z = 0.19 (P = 0.85)						
**1.4.4 Any AE grade3+**						
RTOG 9601	8	382	17	374	47.9%	0.46 [0.20, 1.05]
SPPORT	41	563	18	547	52.1%	2.21 [1.29, 3.80]
**Subtotal (95% CI)**		**945**		**921**	**100.0%**	**1.04 [0.22, 4.85]**
Total events	49		35			
Heterogeneity: Tau² = 1.10; Chi² = 9.67, df = 1 (P = 0.002); I² = 90%						
Test for overall effect: Z = 0.05 (P = 0.96)						

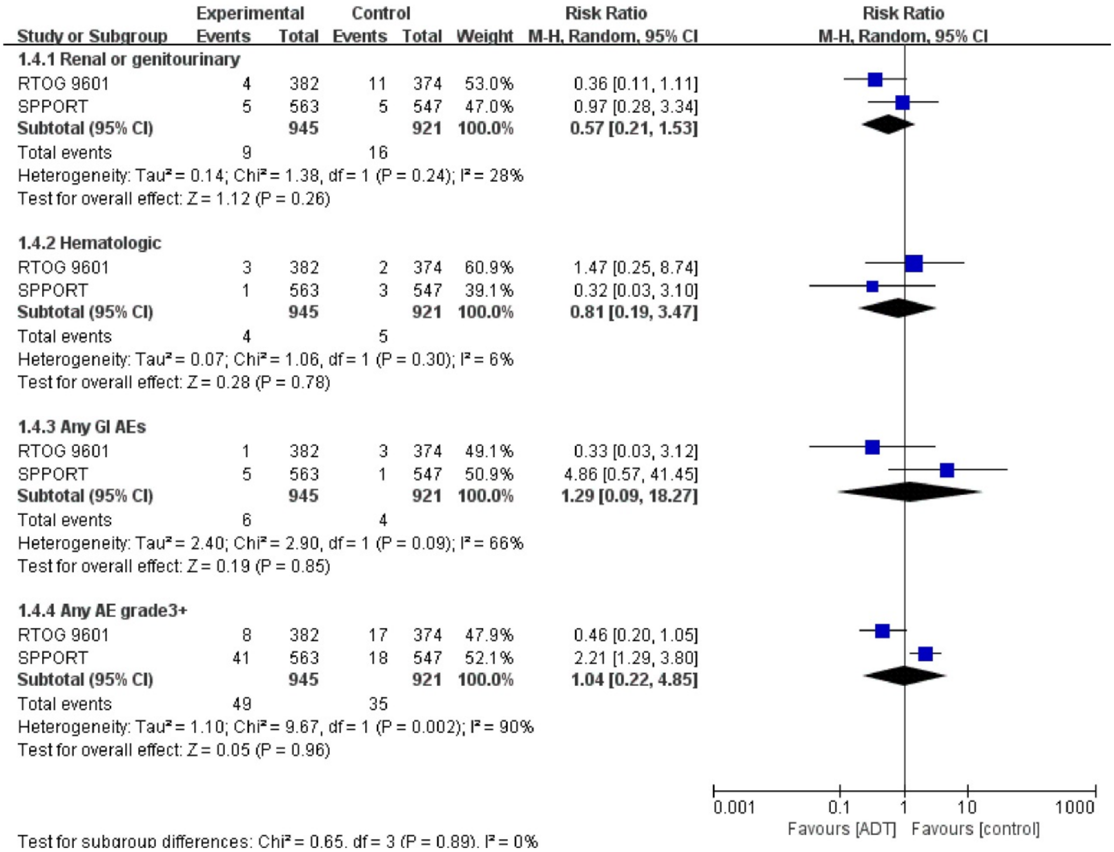

Test for subgroup differences: Chi² = 0.65, df = 3 (P = 0.89), I² = 0%

Abbreviations: The diamond indicates best estimate of the true (pooled) outcome (with width indicating 95% CI). RR, risk ratio; experimental stands for salvage radiotherapy combined with endocrine therapy; control stands for salvage radiotherapy alone. Since there is no heterogeneity, a random-effects model is used.

eFigure 5. Assessment of Late Adverse Events

Study or Subgroup	Experimental Events	Experimental Total	Control Events	Control Total	Weight	Risk Ratio M-H, Fixed, 95% CI
**1.5.1 Renal or genitourinary**						
GETUG-AFU 16	26	366	29	372	34.1%	0.91 [0.55, 1.52]
RTOG 9601	27	382	26	374	31.1%	1.02 [0.60, 1.71]
SPPORT	37	559	29	545	34.8%	1.24 [0.78, 1.99]
**Subtotal (95% CI)**		**1307**		**1291**	**100.0%**	**1.06 [0.80, 1.41]**
Total events	90		84			
Heterogeneity: Chi² = 0.81, df = 2 (P = 0.67); I² = 0%						
Test for overall effect: Z = 0.40 (P = 0.69)						
**1.5.2 Hematologic**						
RTOG 9601	6	382	4	374	57.1%	1.47 [0.42, 5.16]
SPPORT	2	559	3	545	42.9%	0.65 [0.11, 3.87]
**Subtotal (95% CI)**		**941**		**919**	**100.0%**	**1.12 [0.41, 3.07]**
Total events	8		7			
Heterogeneity: Chi² = 0.54, df = 1 (P = 0.46); I² = 0%						
Test for overall effect: Z = 0.22 (P = 0.83)						
**1.5.3 Any GI AEs**						
GETUG-AFU 16	6	366	5	372	30.8%	1.22 [0.38, 3.96]
RTOG 9601	11	382	7	374	44.0%	1.54 [0.60, 3.93]
SPPORT	5	559	4	545	25.2%	1.22 [0.33, 4.51]
**Subtotal (95% CI)**		**1307**		**1291**	**100.0%**	**1.36 [0.72, 2.58]**
Total events	22		16			
Heterogeneity: Chi² = 0.13, df = 2 (P = 0.94); I² = 0%						
Test for overall effect: Z = 0.94 (P = 0.35)						
**1.5.4 Sexual disorder**						
GETUG-AFU 16	30	366	20	372	55.1%	1.52 [0.88, 2.63]
RTOG 9601	28	382	16	374	44.9%	1.71 [0.94, 3.11]
**Subtotal (95% CI)**		**748**		**746**	**100.0%**	**1.61 [1.08, 2.41]**
Total events	58		36			
Heterogeneity: Chi² = 0.08, df = 1 (P = 0.78); I² = 0%						
Test for overall effect: Z = 2.31 (P = 0.02)						
**1.5.5 Any AE grade3+**						
RTOG 9601	100	382	73	374	52.8%	1.34 [1.03, 1.75]
SPPORT	87	559	65	545	47.2%	1.30 [0.97, 1.76]
**Subtotal (95% CI)**		**941**		**919**	**100.0%**	**1.32 [1.09, 1.62]**
Total events	187		138			
Heterogeneity: Chi² = 0.02, df = 1 (P = 0.89); I² = 0%						
Test for overall effect: Z = 2.77 (P = 0.006)						

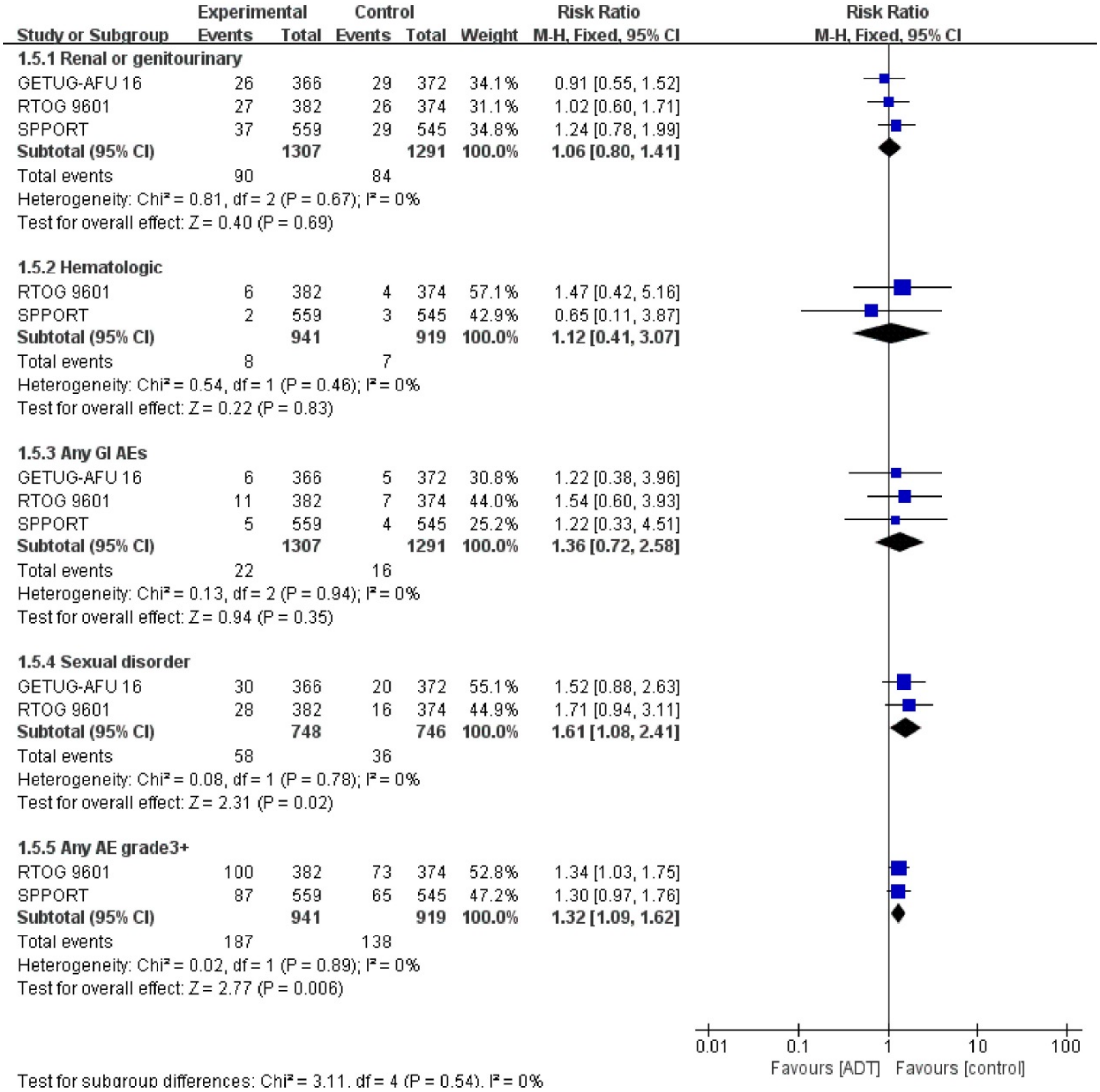

Test for subgroup differences: Chi² = 3.11, df = 4 (P = 0.54), I² = 0%

Abbreviations: The diamond indicates best estimate of the true (pooled) outcome (with width indicating 95% CI). RR, risk ratio; experimental stands for salvage radiotherapy combined with endocrine therapy; control stands for salvage radiotherapy alone. Since there is no heterogeneity, a fixed-effects model is used.

eFigure 6. Funnel Plot of biochemical Progression Free Survival.

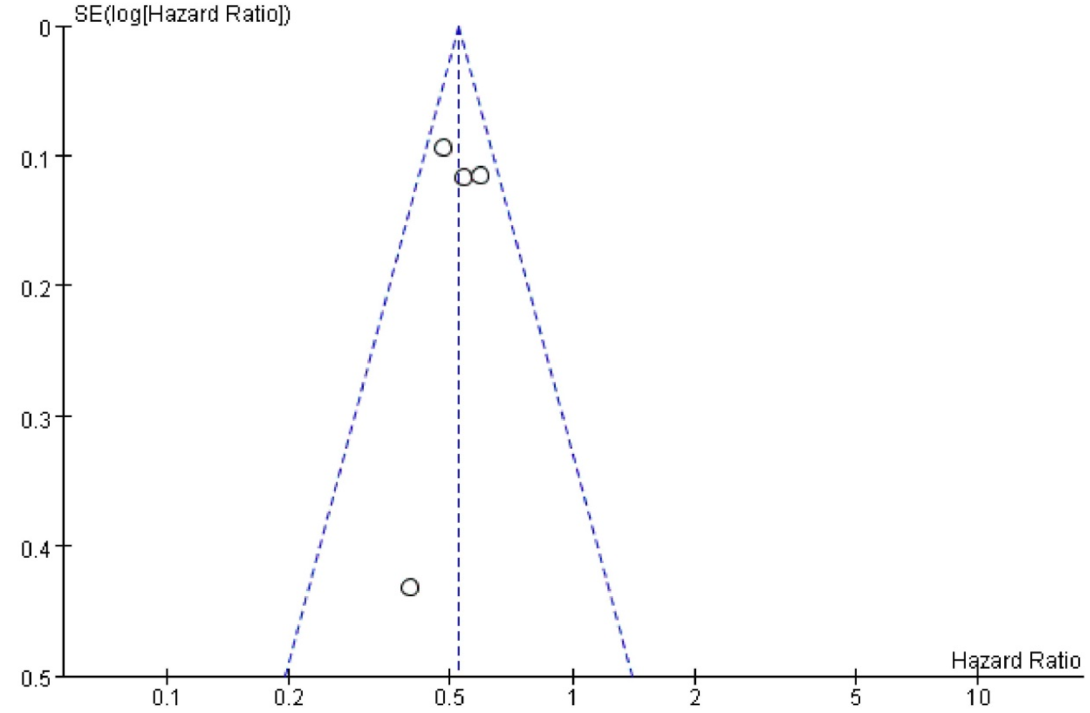

eFigure 7. Funnel Plot of Metastasis Free Survival.

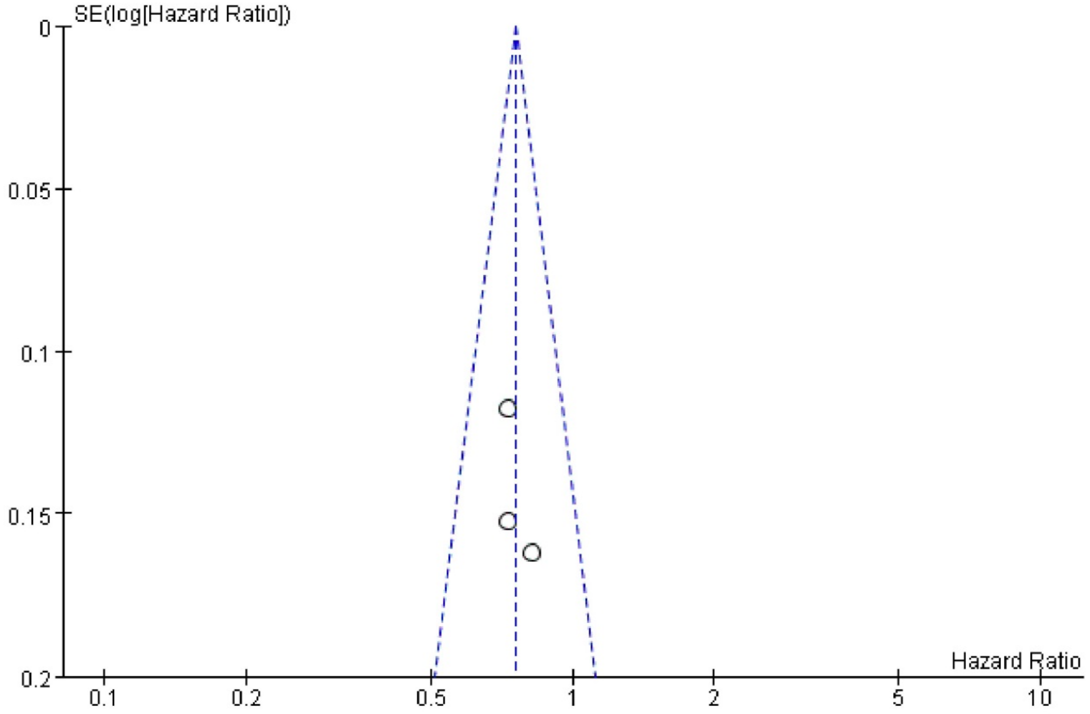

eFigure 8. Funnel Plot of Overall Survival.

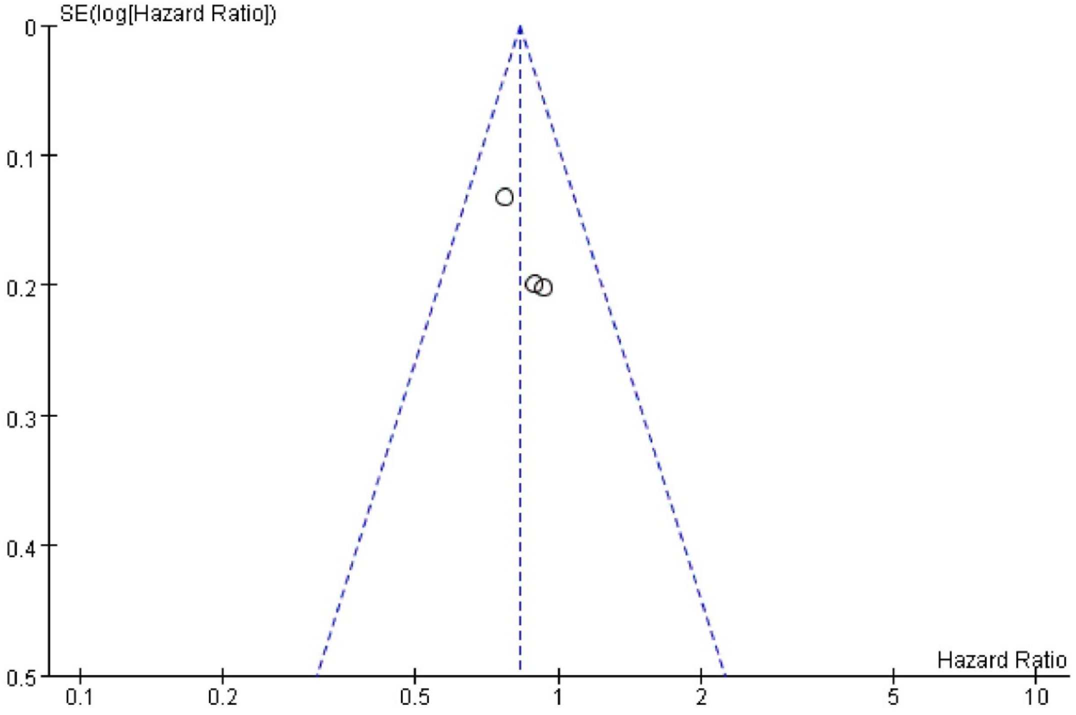

eMethods. Search Strategy

PubMed <2012-01-01 to 2022-10-10> Search Strategy (918)

#1 "Radiotherapy"[Mesh]

#2 (Radiotherapies) OR (Radiation Therapy) OR (Radiation Therapies) OR (Therapies, Radiation) OR (Therapy, Radiation) OR (Radiation Treatment) OR (Radiation Treatments) OR (Treatment, Radiation) OR (Radiotherapy, Targeted) OR (Radiotherapies, Targeted) OR (Targeted Radiotherapies) OR (Targeted Radiotherapy) OR (Targeted Radiation Therapy) OR (Radiation Therapies, Targeted) OR (Targeted Radiation Therapies) OR (Therapies, Targeted Radiation) OR (Therapy, Targeted Radiation) OR (Radiation Therapy, Targeted)

#3 #1 OR #2

#4 "Androgen Antagonists"[Mesh]

#5 "Androgen Receptor Antagonists"[Mesh]

#6 "Nonsteroidal Anti-Androgens"[Mesh]

#7 "Hormone Replacement Therapy"[Mesh]

#8 (Therapy, Hormone Replacement) OR (Hormone Replacement Therapies) OR (Replacement Therapies, Hormone) OR (Therapies, Hormone Replacement) OR (Replacement Therapy, Hormone) OR (androgen deprivation therapy) OR (antiandrogen therapy) OR (cancer hormone therapy) OR (hormone substitution) OR (deprivation therapy) OR (ADT) OR (Androgen Deprivation Treatments) OR (castration) OR (androgen deprivation therapy)

#9 #4 OR #5 OR #6 OR #7 OR #8

#10 "Prostatic Neoplasms"[Mesh]

#11 (Prostate Neoplasms) OR (Neoplasms, Prostate) OR (Neoplasm, Prostate) OR (Prostate Neoplasm) OR (Neoplasms, Prostatic) OR (Neoplasm, Prostatic) OR (Prostatic Neoplasm) OR (Prostate Cancer) OR (Cancer, Prostate) OR (Cancers, Prostate) OR (Prostate Cancers) OR (Cancer of the Prostate) OR (Prostatic Cancer) OR

(Cancer, Prostatic) OR (Cancers, Prostatic) OR (Prostatic Cancers) OR (Cancer of Prostate)
#12 #10 OR #11
#13 "Surgical Procedures, Operative"[Mesh]
#14 "surgery" [Subheading]
#15 (Operative Procedures) OR (Operative Procedure) OR (Procedure, Operative) OR (Procedures, Operative) OR (Surgical Procedure, Operative) OR (Operative Surgical Procedures) OR (Procedure, Operative Surgical) OR (Procedures, Operative Surgical) OR (Surgical Procedures) OR (Procedure, Surgical) OR (Procedures, Surgical) OR (Surgical Procedure) OR (Operative Surgical Procedure) OR (Surgery, Ghost) OR (Ghost Surgery) OR (radical prostatectomy)
#16 #13 OR #14 OR#15
#17 "Recurrence"[Mesh]
#18 (biochemical recurrence) OR (recurrence) OR (rising prostate specific antigen) OR (PSA recurrent)
#19 #17 OR #18
#3 AND #9 AND #12 AND #16 AND #19

Embase <2012-01-01 to 2022-10-10> Search Strategy（2771）
#1 'radiotherapy'/exp
#2 'radiation'/exp
#3 radiotherapies:ab,ti OR 'radiation therapy':ab,ti OR 'radiation therapies':ab,ti OR 'therapies, radiation':ab,ti OR 'therapy, radiation':ab,ti OR 'radiation treatment':ab,ti OR 'radiation treatments':ab,ti OR 'treatment, radiation':ab,ti OR 'radiotherapy, targeted':ab,ti OR 'radiotherapies, targeted':ab,ti OR 'targeted radiotherapies':ab,ti OR 'targeted radiotherapy':ab,ti OR 'targeted radiation therapy':ab,ti OR 'radiation therapies, targeted':ab,ti OR 'targeted radiation therapies':ab,ti OR 'therapies, targeted radiation':ab,ti OR 'therapy, targeted radiation':ab,ti OR 'radiation therapy, targeted':ab,ti
#4 #1 OR #2 OR #3
#5 'antiandrogen'/exp
#6 'androgen receptor antagonist'/exp
#7 'hormonal therapy'/exp
#8 'androgen deprivation therapy'/exp
#9 'therapy, hormone replacement':ab,ti OR 'hormone replacement therapies':ab,ti OR 'replacement therapies, hormone':ab,ti OR 'therapies, hormone replacement':ab,ti OR 'replacement therapy, hormone':ab,ti OR 'antiandrogen therapy':ab,ti OR 'cancer hormone therapy':ab,ti OR 'hormone substitution':ab,ti OR 'deprivation therapy':ab,ti OR adt:ab,ti OR 'androgen deprivation treatments':ab,ti OR castration:ab,ti OR 'androgen deprivation therapy':ab,ti
#10 #5 OR #6 OR #7 OR #8 OR #9
#11 'prostate cancer'/exp
#12 'prostate neoplasms':ab,ti OR 'neoplasms, prostate':ab,ti OR 'neoplasm, prostate':ab,ti OR 'prostate neoplasm':ab,ti OR 'neoplasms, prostatic':ab,ti OR 'neoplasm, prostatic':ab,ti OR 'prostatic neoplasm':ab,ti OR 'prostate cancer':ab,ti OR 'cancer, prostate':ab,ti OR 'cancers, prostate':ab,ti OR 'prostate cancers':ab,ti OR 'cancer of the prostate':ab,ti OR 'prostatic cancer':ab,ti OR 'cancer, prostatic':ab,ti OR 'cancers, prostatic':ab,ti OR 'prostatic cancers':ab,ti OR 'cancer of prostate':ab,ti
#13 #11 OR #12
#14 'surgery'/exp
#15 'prostatectomy'/exp
#16 'operative procedures':ab,ti OR 'operative procedure':ab,ti OR 'procedure, operative':ab,ti OR 'procedures, operative':ab,ti OR 'surgical procedure, operative':ab,ti OR 'operative surgical procedures':ab,ti OR 'procedure, operative surgical':ab,ti OR 'procedures, operative surgical':ab,ti OR 'surgical procedures':ab,ti OR 'procedure, surgical':ab,ti OR 'procedures, surgical':ab,ti OR 'surgical procedure':ab,ti OR 'operative surgical procedure':ab,ti

OR 'surgery, ghost':ab,ti OR 'ghost surgery':ab,ti OR 'radical prostatectomy':ab,ti

#17 #14 OR #15 OR #16

#18 'biochemical recurrence'/exp

#19 'recurrent disease'/exp

#20 'cancer recurrence'/exp

#21 'biochemical recurrence':ab,ti OR recurrence:ab,ti OR 'rising prostate specific antigen':ab,ti OR 'psa recurrent':ab,ti

#22 #18 OR #19 OR #20 OR #21

#4 AND #10 AND #13 AND #17 AND #22

Cochrane library <2012-01-01 to 2022-10-10>（211）

#1 MeSH descriptor: [Radiotherapy] explode all trees

#2 ((Radiotherapies) OR (Radiation Therapy) OR (Radiation Therapies) OR (Therapies, Radiation) OR (Therapy, Radiation) OR (Radiation Treatment) OR (Radiation Treatments) OR (Treatment, Radiation) OR (Radiotherapy, Targeted) OR (Radiotherapies, Targeted) OR (Targeted Radiotherapies) OR (Targeted Radiotherapy) OR (Targeted Radiation Therapy) OR (Radiation Therapies, Targeted) OR (Targeted Radiation Therapies) OR (Therapies, Targeted Radiation) OR (Therapy, Targeted Radiation) OR (Radiation Therapy, Targeted)):ti,ab,kw

#3 #1 OR #2

#4 MeSH descriptor: [Androgen Antagonists] explode all trees

#5 MeSH descriptor: [Hormone Replacement Therapy] explode all trees

#6 ((Therapy, Hormone Replacement) OR (Hormone Replacement Therapies) OR (Replacement Therapies, Hormone) OR (Therapies, Hormone Replacement) OR (Replacement Therapy, Hormone) OR (androgen deprivation therapy) OR (antiandrogen therapy) OR (cancer hormone therapy) OR (hormone substitution) OR (deprivation therapy) OR (ADT) OR (Androgen Deprivation Treatments) OR (castration) OR (androgen deprivation therapy)):ti,ab,kw

#7 #4 OR #5 OR #6

#8 MeSH descriptor: [Prostatic Neoplasms] explode all trees

#9 ((Prostate Neoplasms) OR (Neoplasms, Prostate) OR (Neoplasm, Prostate) OR (Prostate Neoplasm) OR (Neoplasms, Prostatic) OR (Neoplasm, Prostatic) OR (Prostatic Neoplasm) OR (Prostate Cancer) OR (Cancer, Prostate) OR (Cancers, Prostate) OR (Prostate Cancers) OR (Cancer of the Prostate) OR (Prostatic Cancer) OR (Cancer, Prostatic) OR (Cancers, Prostatic) OR (Prostatic Cancers) OR (Cancer of Prostate)):ti,ab,kw

#10 #8 OR #9

#11 MeSH descriptor: [Surgical Procedures, Operative] explode all trees

#12 ((Operative Procedures) OR (Operative Procedure) OR (Procedure, Operative) OR (Procedures, Operative) OR (Surgical Procedure, Operative) OR (Operative Surgical Procedures) OR (Procedure, Operative Surgical) OR (Procedures, Operative Surgical) OR (Surgical Procedures) OR (Procedure, Surgical) OR (Procedures, Surgical) OR (Surgical Procedure) OR (Operative Surgical Procedure) OR (Surgery, Ghost) OR (Ghost Surgery) OR (radical prostatectomy)):ti,ab,kw

#13 #11 OR #12

#14 MeSH descriptor: [Recurrence] explode all trees

#15 ((biochemical recurrence) OR (recurrence) OR (rising prostate specific antigen) OR (PSA recurrent)):ti,ab,kw

#16 #14 OR #15

#3 AND #7 AND #10 AND #13 AND #16

Database	Results	Duplicates	Remaining
PubMed	918	190	728

Embase	2771	248	2523
Cochrane library	211	41	170
Total	3900	479	3421

eReferences

1.Shipley WU, Seiferheld W, Lukka HR, et al. Radiation with or without Antiandrogen Therapy in Recurrent Prostate Cancer. *The New England journal of medicine.* 2017;376(5):417-428. doi:10.1056/ NEJMoa1607529.

2.Jackson WC, Tang M, Schipper MJ, et al. Biochemical Failure Is Not a Surrogate End Point for Overall Survival in Recurrent Prostate Cancer: Analysis of NRG Oncology/RTOG 9601. *Journal of clinical oncology : official journal of the American Society of Clinical Oncology.* 2022;40(27):3172-3179. doi:1 0.1200/JCO.21.02741

3.Carrie C, Magné N, Burban-Provost P, et al. Short-term androgen deprivation therapy combined with radiotherapy as salvage treatment after radical prostatectomy for prostate cancer (GETUG-AFU 16): a 112-month follow-up of a phase 3, randomised trial. *The Lancet Oncology.* 2019;20(12):1740-1749. doi: 10.1016/S1470-2045(19)30486-3.

4.Pollack A, Karrison TG, Balogh AG, et al. The addition of androgen deprivation therapy and pelvic lymph node treatment to prostate bed salvage radiotherapy (NRG Oncology/RTOG 0534 SPPORT): an international, multicentre, randomised phase 3 trial. *Lancet (London, England).* 2022;399(10338):1886-1901. doi: 10.1016/S0140-6736(21)01790-6.

5.Tran PT, Lowe K, Wang H, et al. Phase II, double-blind, randomized study of salvage radiation therapy (SRT) plus enzalutamide or placebo for high-risk PSA-recurrent prostate cancer after radical prostatectomy: The SALV-ENZA Trial. *Journal Clinical Oncology.* 2022;40(16). doi:10.1200/JCO.2022.40.16_suppl.5012
